# Characterization of organoid cultured human breast cancer

**DOI:** 10.1186/s13058-019-1233-x

**Published:** 2019-12-11

**Authors:** Nadine Goldhammer, Jiyoung Kim, Vera Timmermans-Wielenga, Ole William Petersen

**Affiliations:** 1 Department of Cellular and Molecular Medicine, Faculty of Health and Medical Sciences, University of Copenhagen, DK-2200 Copenhagen N, Denmark; 2 Novo Nordisk Foundation Center for Stem Cell Biology (DanStem), Faculty of Health and Medical Sciences, University of Copenhagen, DK-2200 Copenhagen N, Denmark; 3 Pathology Department, Centre of Diagnostic Investigations, Rigshospitalet, DK-2100 Copenhagen Ø, Denmark

**Keywords:** Breast cancer, Organoids, Phenotypic drifting

## Abstract

Organoid cultures are increasingly used to model human cancers experimentally with a view to tailoring personalized medicine and predicting drug responses. Breast cancer is no exception, but in particular, primary breast cancer poses some inherent difficulties due to the frequent presence of residual non-malignant cells in the biopsies. We originally developed an assay for the distinction between malignant and non-malignant structures in primary breast cancer organoid cultures (Petersen et al., Proc Natl Acad Sci (USA) 89(19):9064–8, 1992). Here, we apply this assay to assess the frequency of normal-like organoids in primary breast carcinoma cultures and the cellular composition as a consequence of passaging. We find that in consecutively collected samples of primary human breast cancers, residual non-malignant tissues were observed histologically in five out of ten biopsies. Based on relevant morphogenesis and correct polarization as recorded by expression in luminal epithelial cells of mucin 1 (Muc1), occludin, and keratin 19 (K19) and expression in basal cells of integrin β4, p63, and K14, non-malignant organoids were present in all primary human breast cancer-derived cultures. Furthermore, passaging in a contemporary culture medium was in favor of the selective expansion of basal-like cells. We conclude that organoid cultures of human breast cancers are most representative of the tissue origin in primary culture.

## Introduction

Organoid culture conditions have been devised for multiple human cancer types including those of the colon, esophagus, pancreas, stomach, liver, endometrium, prostate, and breast (for review, see [1]). Recently, a recipe based on a key addition of Neuregulin-1 to the culture medium allowed for the culture of > 80% of human breast cancer biopsies. In a preliminary characterization of the breast cancer organoids, some were passaged for > 20 passages [2]. A total of 95 “lines” were obtained, but in general, the identity of the cultured cells was not obvious. Previous attempts to, for example, predict drug response by using 2D or 3D primary cell culture models of cancer have been complicated by the potential concurrent growth of cells from residual, non-malignant tissue ([3, 4]; for reviews, see [5–7]). As far as breast cancer is concerned, a solution to this problem was offered by defining the reproducible behavior of normal-like and non-malignant cells in 3D organoid assays ([8–10]; for review, see also [11]). Here, we use this assay to assess how often non-malignant tissue contributes to organoid culture within a sample of primary breast cancer and how this affects the outcome of the culture if passaged.

## Methods

### Tissue collection

Normal breast biopsies were collected from seven healthy women undergoing reduction mammoplasty for cosmetic reasons and ten women undergoing mastectomy for primary breast cancer. Donors were informed before the surgery and agreed by written consent to donate tissues. The use of human material has been reviewed by the Regional Scientific Ethical Committees (Region Hovedstaden, with reference to H-2-2011-052, H-2-2010-051, and H-3-2010-095). The permit for obtaining clinical material did not include access to basic information regarding patients, and their detailed medical histories are not given to the authors. Normal breast biopsies were processed and frozen as organoids as previously described [12], while tumor biopsies were processed immediately upon receipt and cultured as described below.

### Organoid cultures

To obtain organoid cultures from normal myoepithelial or luminal cell populations (*n* = 4 biopsies), frozen organoids were thawed, trypsinized, and sorted as previously described [13]. For single-cell plating, cells were FACS-sorted using antibodies against the epithelial marker Trop2 and the myoepithelial marker CD271 (modified from [13]) (Additional file 1). Freshly sorted luminal and myoepithelial cells were counted, and 5 × 10^4^ cells were embedded in 100 μl ice-cold Matrigel (Cultrex growth factor reduced BME type 2, Trevigen) in 24-well plates. After solidification of the Matrigel, 350 μl breast cancer organoid medium ([2] and Additional file 2) was added, and cells were kept at 37 °C and 5% CO_2_. The medium was changed every 3–4 days.

For intact organoids, we used either fresh tissue or freshly thawed organoids which were first pipetted up and down repeatedly to reduce the size as described in [2]. Next, these untrypsinized organoids were embedded in 30 or 80 μl Matrigel depending on the tumor volume. After solidification of the Matrigel-cell solution in 24-well plates, breast cancer organoid medium was added [2] and the medium was changed every 4 days. The medium composition as well as information on the culture conditions is summarized in Additional files 2 and 3. Organoids were harvested for immunostaining after 2 to 4 weeks. Organoids were trypsinized and passaged approximately every 14–21 days.

### Immunohistochemistry

Organoid cultures were frozen in n-hexane and mounted for cryostat sections (6–8 μm). Cryostat sections were prepared and stained by immunofluorescence as previously described [13] (Additional file 1). The staining results were quantified either by manual cell counting or by automatic cell counting using the open-source image processing program, Fiji [14].

### Statistical analyses

Percentages of K19^+^ and K14^+^ cells in the cryostat sections from primary biopsies were assessed by measuring four different, randomly chosen areas of K19 and K14 staining, respectively, using Fiji and normalizing to the total cell numbers by dividing it with the area of the nuclear stain in the same area. To assess the numbers of malignant vs non-malignant organoids in organoid cultures, these organoids were counted manually in stained organoid sections (> 5 cells per organoid and 4 to 56 organoids per section). Organoids were counted as non-malignant if they showed polarized K14^+^/K19^+/−^ or integrin β4^+^/Muc1^+/−^ staining, while malignant organoids were those that were only K19^+^ or Muc1^+^. The statistical analysis, including normal distribution and *t* test, was performed by a statistical analysis program R (version 3.5.3).

### Whole-genome sequencing

Genomic DNA was isolated from three pairs of primary tumor samples and their organoids in passages 3 and 4, using the DNeasy Blood and Tissue kit (Qiagen). Whole-genome sequencing and its bioinformatics analysis were performed by BGI Tech Solutions (Hong Kong). In short, 1 μg Genomic DNA per sample was used for short-insert fragment (an average 350 bps) library preparation with BGISEQ in-house master mix. The libraries were sequenced with pair-end 150 bp runs using the BGISEQ-500 platform, and high-throughput sequencing was performed in each library with 30× depth per sample. Raw image files were processed by BGISEQ-500 base-calling software and Genome analysis Toolkit (GATK) (https://www.broadinstitute.org/gatk/guide/best-practices) for variant analysis [15]. Total clean data were mapped to the human reference genome GRCh37/HG19, using Burrows-Wheeler Aligner [16]. On average, 99.9% mapped successfully and 92% mapped uniquely with an average 44-fold sequencing depth on the whole genome.

Local realignment around indels and base quality score recalibration were performed using GATK with duplicate reads removed by Picard tools (http://broadinstitute.github.io/picard/). The sequencing depth and coverage for each individual were calculated based on the alignments. The genomic variations, including single nucleotide polymorphisms (SNPs) and indels, were detected by HaplotypeCaller of GATK (v3.3.0). The variant quality score recalibration (VQSR) method, which uses machine learning to identify the annotation profiles of variants that are likely to be real, was applied to get high-confident variant calls. The copy number variants (CNVs) were detected using the CNVnator (v0.2.7), a read-depth algorithm [17]. IGV (https://software.broadinstitute.org/software/igv/), Gitools (http://www.gitools.org/), and Clinvar (https://www.ncbi.nlm.nih.gov/clinvar/) programs were used for the visualization of genes commonly mutated in breast cancer (COSMIC, https://cancer.sanger.ac.uk/cosmic [18]).

## Results

### Behavior of normal myoepithelial cells and luminal epithelial cells in organoid culture

**Fig. 1:**
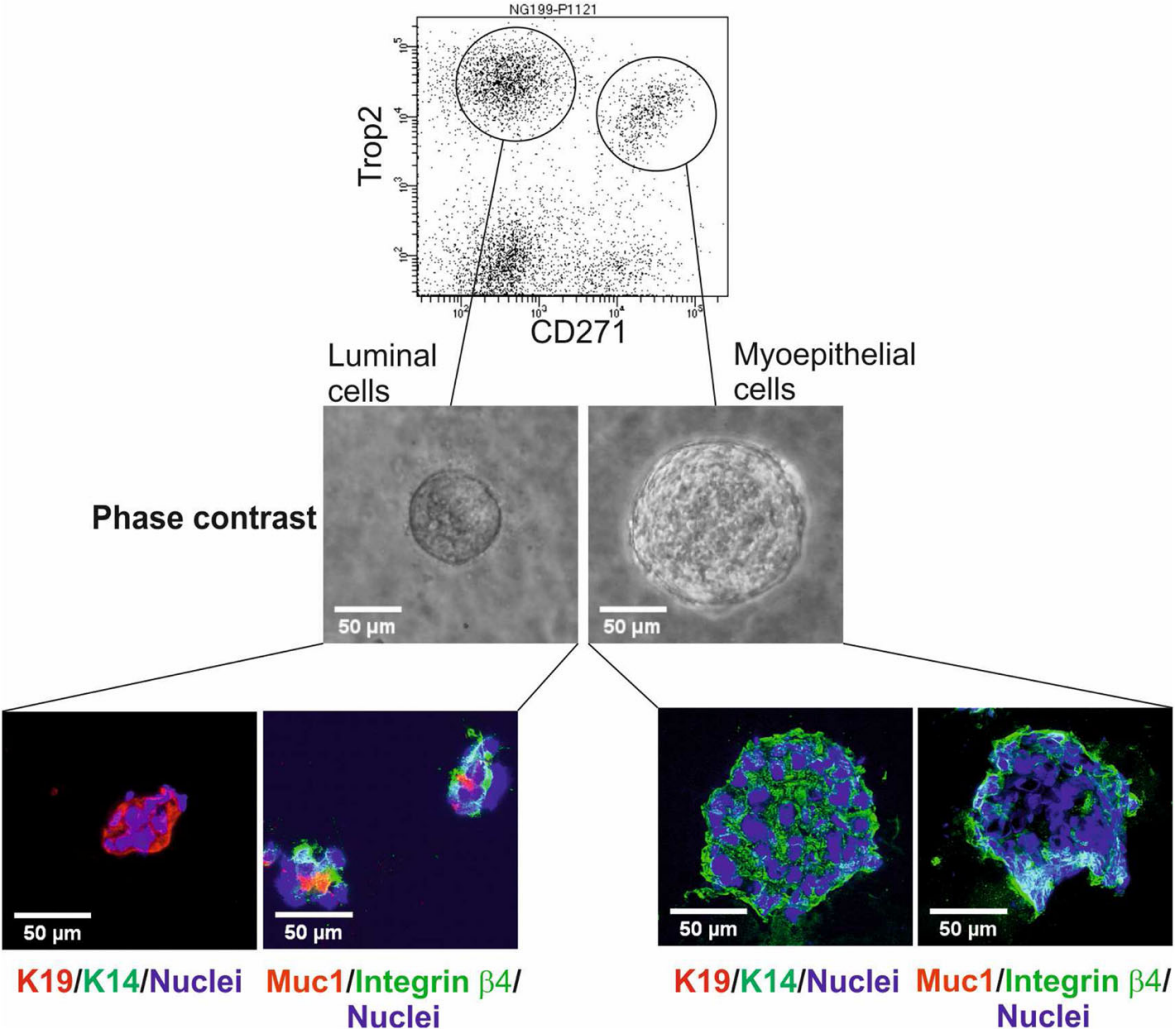
Organoid cultures derived from luminal and myoepithelial cells give rise to lineage-restricted organoids. (Top) FACS diagram of the sorting strategy leading to isolation of pure luminal epithelial and myoepithelial cell populations using epithelial marker Trop2 and myoepithelial marker CD271. (Middle) Phase-contrast micrographs of representative primary normal breast organoids from luminal epithelial and myoepithelial cells. A total of 79 and 90 organoids derived from luminal and myoepithelial cells were evaluated (*n* = 4 biopsies). (Bottom) Representative fluorescence micrographs of cryostat sections from luminal- (left) and myoepithelial-derived organoids (right) stained for either K19-AF568, K14-AF488, and DAPI or Muc1-AF568, integrin β4-AF488, and DAPI

We first examined how normal human breast epithelial cells from four different reduction mammoplasties responded to a breast cancer culture medium containing the essential components of Neuregulin-1 and the ROCK inhibitor Y-27632 [2]. For this purpose, we initially aimed for optimal separation of normal luminal epithelial and myoepithelial cells by a FACS-based protocol relying on a combination of antibodies against the EpCAM family-related trophoblast surface antigen 2, Trop2, and the nerve growth factor receptor, CD271 (Fig. 1). Upon plating inside the breast cancer organoid assay as single cells, luminal cells grew up to form approximately 50-μm-sized acinus-like structures before growth arrest while myoepithelial cells formed larger ball-like structures, both reminiscent of what has been described originally with another culture medium (Fig. 1 and [8]). To provide further evidence for the presence of non-malignant cells in the organoid assay, we sectioned and stained the gels with markers of polarity and basement membrane deposition. As seen in Fig. 1, while the acinus-like luminal-derived structures resembled correctly polarized acini with Muc1 expressed towards a central lumen, both luminal and myoepithelial structures stained with β4 integrin at the cell-extracellular matrix (ECM) junction—a hallmark of non-malignant behavior (Fig. 1 and [8]). This is in contrast to malignant lesions. Thus, while about 50% of basal-like breast cancers exhibit staining with β4 integrin, this is focal and mostly unpolarized [19].

**Fig. 2:**
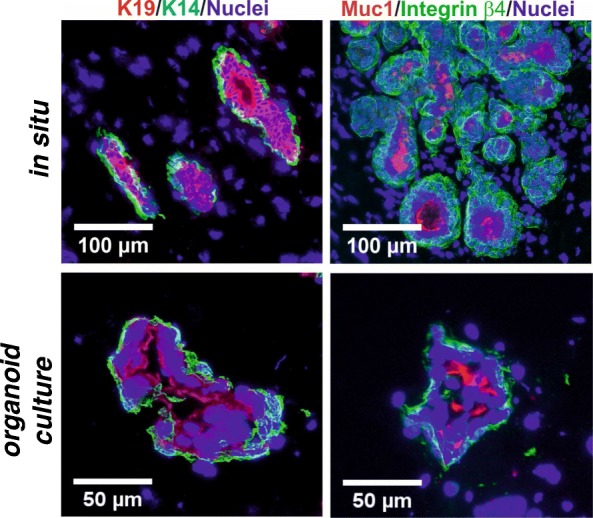
Normal-derived intact organoids in culture resemble in situ normal breast biopsies. Representative fluorescence micrographs of cryostat sections from normal breast biopsies (top) and normal breast organoids (bottom) stained for either K19-AF568, K14-AF488, and DAPI (left) or Muc1-AF568, integrin β4-AF488, and DAPI (right). Note that organoids in the culture retain a double-layered organization

Since primary culture organoid assays often rely on embedding untrypsinized organoids, we also examined how luminal epithelial and myoepithelial cells behaved if not trypsinized and sorted prior to plating. As seen in Fig. 2, under this condition, the normal organoids remained correctly polarized and organized in a double-layered manner reminiscent of this in vivo organization. Even if left to grow for weeks in culture as mainly conveyed by the outer layer of cells, this bi-layered configuration remained intact in all profiles examined and as such remained a breast-specific behavior of non-malignant organoids.

### Breast cancer-derived organoid cultures

**Fig. 3:**
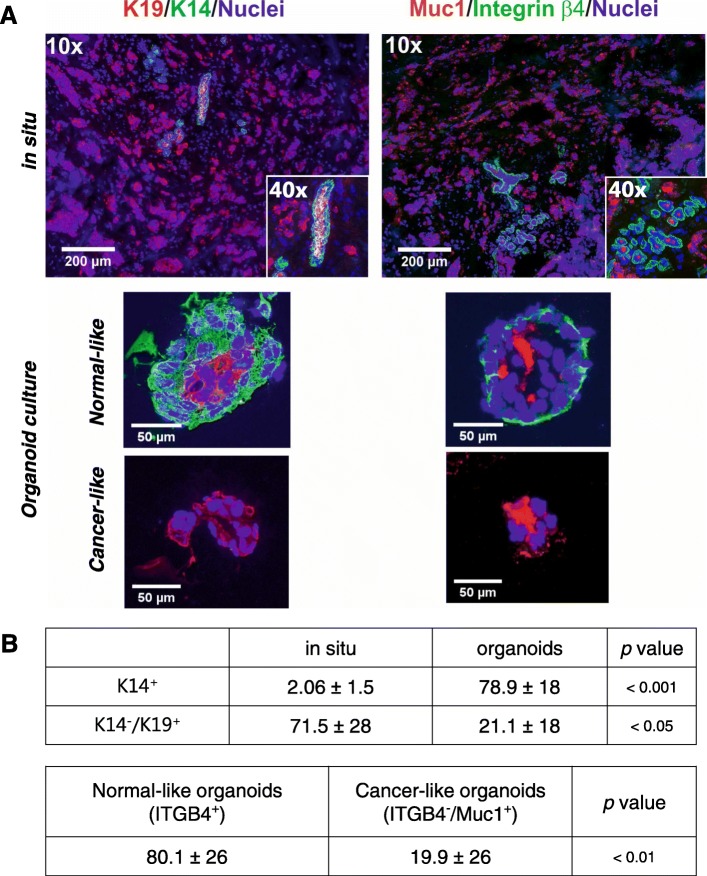
Tumor-derived primary organoid cultures consist of normal-like and cancer-like organoids. **a** Representative fluorescence micrographs of cryostat sections from breast cancer biopsies in situ (top) and corresponding representative tumor-derived organoids (bottom) stained for either K19-AF568, K14-AF488, and DAPI (left) or Muc1-AF568, integrin β4-AF488, and DAPI (right). **b** Quantification of immunostaining of in situ profiles and organoids. (Top) While in situ K14^+^ cells are relatively rare and almost always part of non-malignant profiles, they dominate in derived organoid cultures. The opposite relation is seen as far as K19^+^ only cells are concerned. Data are presented as the mean percentage ± SD of the areas positive for K14 or K19 in situ normalized by nuclear staining or the mean percentage ± SD of organoid numbers positive for K14 or K19. (Bottom) Comparison of the numbers of organoids positive for integrin β4 (ITGB4) vs Muc1. Data are presented as the mean percentage ± SD of the organoid numbers. Five independent tumor samples are examined for the analysis, and indicated *p* values are tested by *t* test

In order to distinguish breast cancer-derived organoids from normal breast organoids in culture, we took advantage of the unique breast-specific pattern described above for normal cells; that by definition, non-malignant/benign epithelial components comprise an outer layer of K14 and integrin β4-positive myoepithelial cells. In contrast, essentially, all nests of malignant cells as they present themselves in situ are devoid of such a layer [4]. By using this criterion and based on staining of multiple sections, we found that non-malignant epithelial profiles comprised only a minority (2.06 ± 1.5%) as compared to malignant cells (79.6 ± 37%, *p* < 0.01) (Fig. 3a, upper panel of in situ). As inferred by Sachs et al. [2], primary organoid culture of such carcinomas indeed captured the mixed composition of the tumor of origin here demonstrated by the presence of both double-layered non-malignant-like structures as well as nests of unpolarized tumor-like cells devoid of myoepithelial cells (Fig. 3a, lower panel of organoid culture). This observation was further substantiated by apical staining with ZO-1 and occludin against tight junction proteins, which is either absent or perturbed in cancer ([10], reviewed in [20]). Furthermore, we found that the specific myoepithelial marker p63, which is very rare in breast cancer, clearly stained some organoids (Additional file 4). In contrast, the estrogen receptor, which is often present in primary tumors, did not stain in organoids (data not shown). At the end of the primary culture period (2 to 4 weeks), the cultures consisted of both cancer-like and normal-like/benign profiles (78.9% non-malignant-like profiles, *p* < 0.001) (Fig. 3b). This was interpreted in favor of all primary carcinomas containing non-malignant, residual components albeit in such relatively low numbers that they sometimes escape in situ histological identification.

**Fig. 4:**
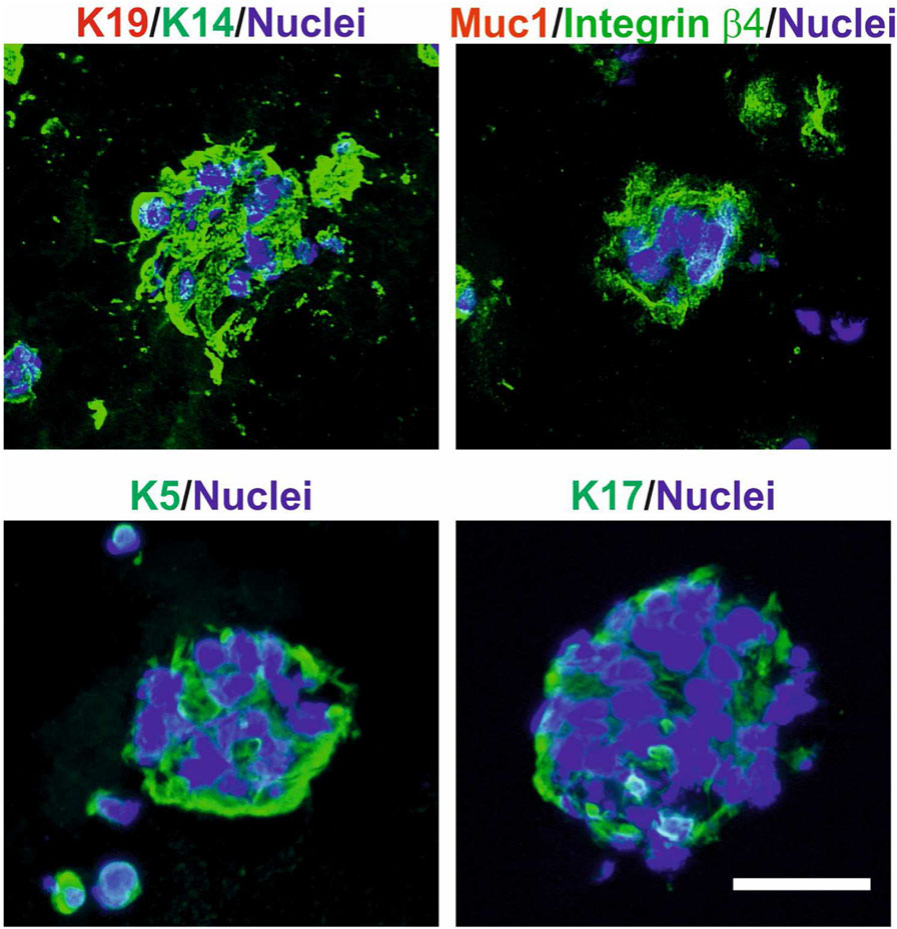
K14^+^/ITGB4^+^ cells dominate in tumor-derived organoid cultures after passaging. Representative fluorescence micrographs of cryostat sections from tumor-derived organoids in passage 2 or 3 stained for K19-AF568/K14-AF488 (upper left), Muc1-AF568/integrin β4-AF488 (upper right), K5-AF488 (lower left), and K17-AF488 (lower right). The nuclei were stained with DAPI. Scale bar, 50 μm

**Fig. 5:**
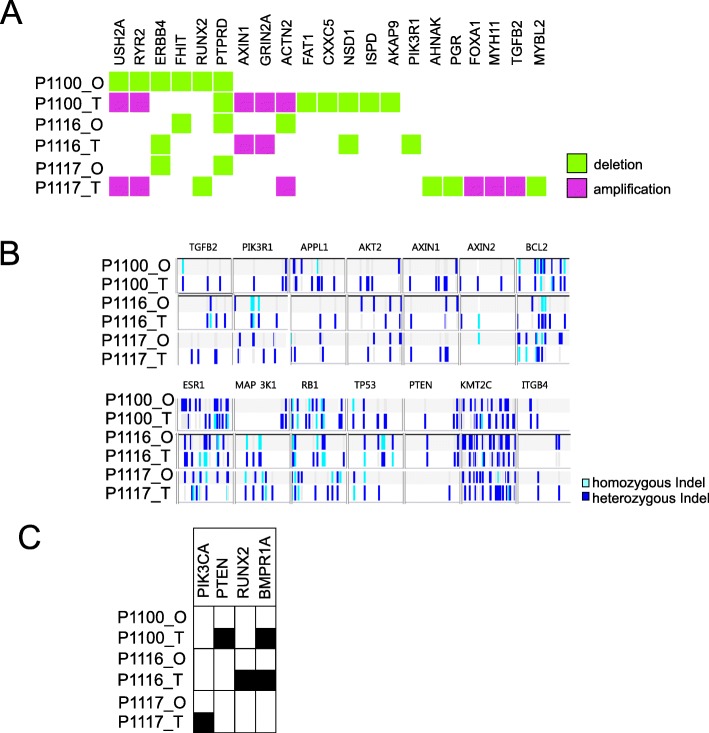
Genomic aberrations in primary tumors and corresponding organoids. Representations of copy number variations (**a**), indels (**b**), and clinically relevant SNPs (**c**) of driver oncogenes and tumor suppressor genes relevant to breast cancer. Genomic DNAs of primary tumors (T) and corresponding organoids (O) from three different patients (P1100, P1116, P1117) were analyzed by whole-genome sequencing (detailed information in the “Methods” section). Represented SNPs (black boxes) are PIK3CA (RCV000024623.6, RCV000154512.1, RCV000201232.1), PTEN (RCV000008256.2, RCV000008257.2, RCV000128455.2, RCV000162649.3, RCV000212882.1), RUNX2 (RCV000177104.2), and BMPR1A (RCV000034703.1, RCV000120253.2, RCV000131909.2). Note that organoids show different or much less genomic aberrations compared to primary tumors

Since in primary culture luminal cells remained K19^+^ and K14^−^, we used this criterion to also identify cancer cells upon passaging in organoid cultures. We passaged the primary organoids by trypsinization into single cells after 2 weeks and kept them for another additional 2 weeks in the second passage. Some were passaged further to third and fourth passages. Indeed, the assay supported the growth of single cells recovered from the trypsinization of primary organoids. Thus, the second to fourth passage organoids grew up to form solid ball-like structures. However, staining of K14 and integrin β4 as well as other myoepithelial/basal keratins K5 and K17 revealed a composition similar to that of the basal layer of myoepithelial cells as described above from flow-sorted reduction mammoplasties (Fig. 4). In none of the passaged organoid cultures, the growth of luminal-like cells was recorded by the staining criteria used (Fig. 4). These data are in favor of some drifting of the collective phenotype of the culture. Whether this is echoed in the overall genomic drifting was assessed by whole-genome sequencing of three paired samples of tumors and third/fourth cultures. As seen in Fig. 5, the landscape of copy number variations among the selected candidate breast cancer driver genes in general underwent a change towards a dilution of the in vivo genomic aberrations. This can only arise as a consequence of a selection of less aberrant cells during organoid cultures. That these nevertheless exhibit substantial genomic copy number alterations is not incompatible with a potential non-malignant or benign origin from uninvolved breast tissue of breast carcinomas [21]. Since most breast cancer predisposition loci are present already at the level of ductal carcinomas in situ (DCIS [22];), we also screened for breast cancer-associated single nucleotide variations. Indeed, the fact that we find examples of culture-induced “normalizations” of single nucleotide variations suggests the selection in the culture of clones upstream of DCIS cells (Fig. 5).

Collectively, we conclude that organoid cultures of human breast cancer drift upon passaging of the cells which may confound predictions based on drug screens in such cultures.

## Discussion

Here, we reappraise the complicated technology of organoid culturing of human breast cancer by combining early measures to identifying the organoids with a contemporary recipe for long-term culture. We provide evidence that fidelity in terms of resemblance between tissue and culture is highest in the early passage. In the present study, we failed to establish permanent cell lines from primary human breast cancer even with this new recipe. This may be because our material was limited to 10 biopsies. We and others, however, have previously shown that successful long-term culture of human breast cancer is a relatively rare event in the order of 1 out of 30 biopsies [4, 23]. Most success is obtained with basal-like breast cancer-derived cultures and certain metastatic lesions. This appears also to apply to the new recipe medium relying on a large material where those of the organoid cultures qualifying for the in vivo drug testing were all grade 3 ER-negative (10 T), basal-like derived (27 T), or from metastatic lesions (33 T and 213 T) [2].

However, even more importantly, what contributes to the complexity in particular of primary breast cancer organoid culture is the almost omnipresence of residual, non-malignant tissue. In this respect, it is worthwhile noticing that inclusion of Neuregulin-1 in the medium is probably not the answer to specific growth requirements of cancer cells. Rather, Neuregulin-1 has been shown to extend the growth of normal mammary organoids [24].

It could be argued that the same markers that we use here to claim non-malignancy, e.g., β4 integrin and keratin K5, in a cancer context are used as markers of subsets of cancer cells with EMT or stem-like properties [19, 25]. However, β4 integrin stains one third of breast cancer biopsies only [19], and we had no basal-like breast cancers stained with β4 integrin among tumor cells in our sample. We further note that unlike the typical basal staining that we see in non-malignant tissue, when seen at the surface of cancer cells, β4 integrin is very rarely polarized correctly [19]. Therefore, our observation of a strong polarized staining at the cell-ECM junction of the organoids in passaged cultures is in favor of a non-malignant or benign origin. A similar logic is applicable to staining with the two other markers keratin K5 and K17. Thus, in contrast to the uniform staining of non-malignant tissue, when present in basal-like cancer, K5 and K17 mostly appear in scattered single cells. Accordingly, the widespread staining with basal keratins in profiles of the cultured organoids points towards a non-malignant origin.

Finally, it is important to appreciate the limitations of genomics in deciding between malignant and non-malignant since apparently uninvolved breast tissue from breast cancer patients shows many of the same aberrations as the cancer itself [21], and the majority of premalignant lesions resemble invasive cancer by the mutational profile [22].

Collectively, these considerations underscore that using human breast organoid culture for clinical purposes should if at all be restricted to primary culture.

## Supplementary information

**Additional file 1.** Overview of antibodies used. Table showing antibodies and their dilutions used for FACS and immunohistochemistry

**Additional file 2.** Reagents and medium composition used. Table showing reagents used and composition of breast cancer organoid medium including variations from the original recipe by Sachs et al. [2]

**Additional file 3.** Overview of breast cancer biopsies and breast cancer-derived organoid cultures included. Table showing information about breast cancer biopsies and corresponding organoid cultures

**Additional file 4.** Primary organoid culture is most representative of the tumor of origin. Representative fluorescence micrographs of cryostat sections from breast cancer biopsies in situ (top) and corresponding representative tumor-derived organoids (middle and bottom) stained for either K19-AF568, ZO-1-AF488, and DAPI (left), K19-AF568, Occludin-AF488, DAPI (middle) or p63-AF568, K14-AF488 and DAPI (right). Arrows indicate residual normal-like structures in breast cancer biopsies. Note that normal-like structures are not obvious in situ in two of the biopsies in spite of the presence of normal-like organoids in the corresponding cultures

## Data Availability

The datasets used and/or analyzed during the current study are available from the corresponding author on reasonable request.

## Abbreviations

ECM: Extracellular matrix
K14: Keratin 14
K19: Keratin 19
Muc1: Mucin 1
SNP: Single nucleotide polymorphisms
CNV: Copy number variants
DCIS: Ductal carcinomas in situ

## References

[1] Drost J, Clevers H (2018). Organoids in cancer research. Nat Rev Cancer.

[2] Sachs N, de Ligt J, Kopper O, Gogola E, Bounova G, Weeber F (2018). A living biobank of breast cancer organoids captures disease heterogeneity. Cell..

[3] Petersen OW, van Deurs B (1986). Demonstration of human breast carcinoma cells in cryosections and primary monolayer cultures of surgical biopsies by neotetrazolium reductase cytochemistry. Cancer Res.

[4] Petersen OW, van Deurs B (1987). Preservation of defined phenotypic traits in short-term cultured human breast carcinoma derived epithelial cells. Cancer Res.

[5] Selby PJ, Raghavan D (1981). Role of laboratory chemosensitivity testing in the selection of cancer chemotherapy for individual patients. J Clin Pathol.

[6] Taylor-Papadimitriou J, D’Souza B, Berdichevsky F, Shearer M, Martignone S, Alford D (1993). Human models for studying malignant progression in breast cancer. Eur J Cancer Prev.

[7] Bissell MJ (1981). The differentiated state of normal and malignant cells or how to define a “normal” cell in culture. Int Rev Cytol.

[8] Petersen OW, Ronnov-Jessen L, Howlett AR, Bissell MJ (1992). Interaction with basement membrane serves to rapidly distinguish growth and differentiation pattern of normal and malignant human breast epithelial cells. Proc Natl Acad Sci U S A.

[9] Shearer M, Bartkova J, Bartek J, Berdichevsky F, Barnes D, Millis R (1992). Studies of clonal cell lines developed from primary breast cancers indicate that the ability to undergo morphogenesis in vitro is lost early in malignancy. Int J Cancer.

[10] Pasic L, Eisinger-Mathason TS, Velayudhan BT, Moskaluk CA, Brenin DR, Macara IG (2011). Sustained activation of the HER1-ERK1/2-RSK signaling pathway controls myoepithelial cell fate in human mammary tissue. Genes Dev.

[11] Simian M, Bissell MJ (2017). Organoids: a historical perspective of thinking in three dimensions. J Cell Biol.

[12] Ronnov-Jessen L, Petersen OW (1993). Induction of alpha-smooth muscle actin by transforming growth factor-beta 1 in quiescent human breast gland fibroblasts. Implications for myofibroblast generation in breast neoplasia. Lab Investig.

[13] Fridriksdottir AJ, Villadsen R, Morsing M, Klitgaard MC, Kim J, Petersen OW (2017). Proof of region-specific multipotent progenitors in human breast epithelia. Proc Natl Acad Sci U S A.

[14] Schindelin J, Arganda-Carreras I, Frise E, Kaynig V, Longair M, Pietzsch T (2012). Fiji: an open-source platform for biological-image analysis. Nat Methods.

[15] McKenna A, Hanna M, Banks E, Sivachenko A, Cibulskis K, Kernytsky A (2010). The genome analysis toolkit: a MapReduce framework for analyzing next-generation DNA sequencing data. Genome Res.

[16] Li H, Durbin R (2010). Fast and accurate long-read alignment with Burrows-Wheeler transform. Bioinformatics..

[17] Abyzov A, Urban AE, Snyder M, Gerstein M (2011). CNVnator: an approach to discover, genotype, and characterize typical and atypical CNVs from family and population genome sequencing. Genome Res.

[18] Tate JG, Bamford S, Jubb HC, Sondka Z, Beare DM, Bindal N (2019). COSMIC: the catalogue of somatic mutations in cancer. Nucleic Acids Res.

[19] Lu S, Simin K, Khan A, Mercurio AM (2008). Analysis of integrin beta4 expression in human breast cancer: association with basal-like tumors and prognostic significance. Clin Cancer Res.

[20] Itoh M, Bissell MJ (2003). The organization of tight junctions in epithelia: implications for mammary gland biology and breast tumorigenesis. J Mammary Gland Biol Neoplasia.

[21] Ronowicz A, Janaszak-Jasiecka A, Skokowski J, Madanecki P, Bartoszewski R, Balut M (2015). Concurrent DNA copy-number alterations and mutations in genes related to maintenance of genome stability in uninvolved mammary glandular tissue from breast cancer patients. Hum Mutat.

[22] Petridis C, Brook MN, Shah V, Kohut K, Gorman P, Caneppele M (2016). Genetic predisposition to ductal carcinoma in situ of the breast. Breast Cancer Res.

[23] Petersen OW, van Deurs B, Nielsen KV, Madsen MW, Laursen I, Balslev I (1990). Differential tumorigenicity of two autologous human breast carcinoma cell lines, HMT-3909S1 and HMT-3909S8, established in serum-free medium. Cancer Res.

[24] Jarde T, Lloyd-Lewis B, Thomas M, Kendrick H, Melchor L, Bougaret L (2016). Wnt and Neuregulin1/ErbB signalling extends 3D culture of hormone responsive mammary organoids. Nat Commun.

[25] Bierie B, Pierce SE, Kroeger C, Stover DG, Pattabiraman DR, Thiru P (2017). Integrin-beta4 identifies cancer stem cell-enriched populations of partially mesenchymal carcinoma cells. Proc Natl Acad Sci U S A.

