# Supplementary material for: Characterization of organoid cultured human breast cancer
Source: Breast Cancer Res. 2019 Dec 11;21:141. doi: 10.1186/s13058-019-1233-x (PMC6907265; doi:10.1186/s13058-019-1233-x)
Supplement: Supplementary file 1 — Additional file 1. Overview of antibodies used. Table showing antibodies and their dilutions used for FACS and immunohistochemistry [file 13058_2019_1233_MOESM1_ESM.pdf]

Additional file 1. Overview of antibodies used.

Antibody	Conjugate	Clone	Dilution	Company
**FACS**				
Trop2	PE	162-46	1:50	BD Biosciences
CD271	APC	Me20.4	1:50	Cedarlane Laboratories
**Immunohistochemistry**				
K14	Unconjugated	LL002	1:100	Monosan
K19	Unconjugated	Ba16	1:100	Abcam
K19	Unconjugated	A53-B/A2	1:100	Abcam
Integrinβ4	Unconjugated	3E1	1:500	Chemicon
Muc1	Unconjugated	115D8	1:10	Biogenesis
ZO-1	Unconjugated	ZO1-1A12	1:25	Zymed
Occludin	Unconjugated	18-7431	1:50	Zymed
p63	Unconjugated	7Jul	1:10	Leica
**Secondary antibodies**				
Anti-mouse IgG3	AF488	Goat	1:500	Life Technologies
Anti-mouse IgG1	AF568	Goat	1:500	Life Technologies
Anti-mouse IgG1	AF488	Goat	1:500	Life Technologies
Anti-mouse IgG2b	AF568	Goat	1:500	Life Technologies
Anti-mouse IgG2a	AF568	Goat	1:500	Life Technologies
Anti-rabbit	AF488	Goat	1:500	Life Technologies
