# Supplementary material for: Characterization of organoid cultured human breast cancer
Source: Breast Cancer Res. 2019 Dec 11;21:141. doi: 10.1186/s13058-019-1233-x (PMC6907265; doi:10.1186/s13058-019-1233-x)
Supplement: Supplementary file 2 — Additional file 2. Reagents and medium composition used. Table showing reagents used and composition of breast cancer organoid medium including variations from the original recipe by Sachs et al. [2] [file 13058_2019_1233_MOESM2_ESM.pdf]

Additional file 2. Reagents and medium composition used.

Reagent	Source	Concentration	Cat#
Collagenase	Sigma	1.5 mg·ml^{-1}	C9407
Cultrex growth factor reduced BME type 2	Trevigen	10 mg·ml^{-1}	3533-010-02

Breast Cancer Organoid Medium

Reagent	Source	Concentration	Cat#
Advanced DMEM/F12	Gibco*	1x	21041-025
Gentamycin sulfate	Biological Industries*	50 µg·ml^{-1}	03-035-1B
L-Glutamine	Sigma*	2 mM	G7513
R-Spondin	R&D	250 ng·ml^{-1}	3500-RS/CF
Neuregulin-1	Peprotech	5 nM	100-03
FGF 7	Peprotech	5 ng·ml^{-1}	100-19
FGF 10	Peprotech	20 ng·ml^{-1}	100-26
EGF	Pepreotech	5 ng·ml^{-1}	AF-100-15
Noggin	Peprotech	100 ng·ml^{-1}	120-10C
A83-01	Tocris	500 nM	2939
Y-27632	Axon*	5 µM	1683
SB202190	Sigma	500 nM	S7067
B27	Gibco	1x	17504-44
N-Acetylcysteine	Sigma	1.25 mM	A9165-5g
Nicotinamide	Sigma	5 mM	N0636

* Supplier varies from Sachs et al.
