# Supplementary material for: Characterization of organoid cultured human breast cancer
Source: Breast Cancer Res. 2019 Dec 11;21:141. doi: 10.1186/s13058-019-1233-x (PMC6907265; doi:10.1186/s13058-019-1233-x)
Supplement: Supplementary file 3 — Additional file 3. Overview of breast cancer biopsies and breast cancer-derived organoid cultures included. Table showing information about breast cancer biopsies and corresponding organoid cultures [file 13058_2019_1233_MOESM3_ESM.pdf]

Additional file 3. Overview of breast cancer biopsies and breast cancer-derived organoid cultures included.

Biopsy ID	Source	Type	Number of organoid cultures in 24 wells	Days in culture	Organoids for staining
P1099	Primary BC	Luminal	7	>30	No
P1100	Primary BC	Luminal	8	10	Yes
P1103	Primary BC	Luminal	10	34	Yes
P1104	Primary BC	Basal	7	23	Yes
P1105	Primary BC	Luminal	3	>30	No
P1106	Primary BC	Luminal	6	28	Yes
P1107	Primary BC		6	>30	No
P1109	Primary BC	Basal	6	22	Yes
P1110	Primary BC	Basal	7	>30	No
P821	Lymph Node	Luminal	8	>30	No
