# Supplementary figures and images for: Characterization of organoid cultured human breast cancer
Source: Breast Cancer Res. 2019 Dec 11;21:141. doi: 10.1186/s13058-019-1233-x (PMC6907265; doi:10.1186/s13058-019-1233-x)

***Additional file 4. Primary organoid culture is most representative of the tumor of origin.***

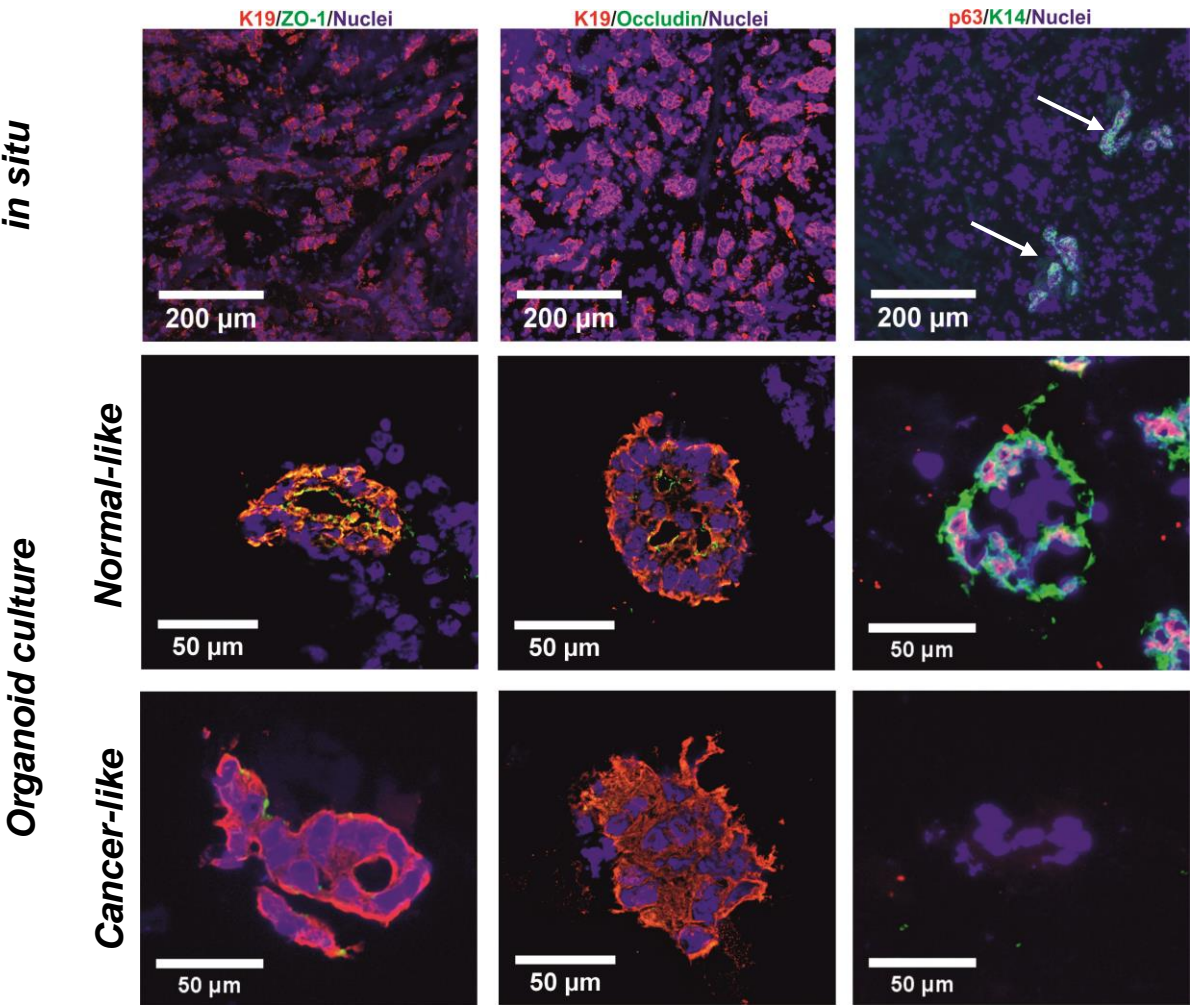

Supplement: Supplementary file 4 — Additional file 4. Primary organoid culture is most representative of the tumor of origin. Representative fluorescence micrographs of cryostat sections from breast cancer biopsies in situ (top) and corresponding representative tumor-derived organoids (middle and bottom) stained for either K19-AF568, ZO-1-AF488, and DAPI (left), K19-AF568, Occludin-AF488, DAPI (middle) or p63-AF568, K14-AF488 and DAPI (right). Arrows indicate residual normal-like structures in breast cancer biopsies. Note that normal-like structures are not obvious in situ in two of the biopsies in spite of the presence of normal-like organoids in the corresponding cultures [file 13058_2019_1233_MOESM4_ESM.pdf]
